# Plasticity in parental care: Interspecific competitor cues shape biparental cooperation in a burying beetle

**DOI:** 10.1111/1365-2656.70251

**Published:** 2026-05-04

**Authors:** Casey Patmore, Per T. Smiseth

**Affiliations:** ^1^ Institute of Ecology and Evolution University of Edinburgh Edinburgh UK

**Keywords:** biparental care, *Calliphora vomitoria*, duration of care, interspecific competition, *Nicrophorus vespilloides*, plasticity

## Abstract

1. Interspecific competition is an important evolutionary driver of many species' life histories and behaviours, arising wherever species come into conflict over limited resources. However, how such competition shapes the plasticity of social behaviours within a species, such as biparental care, remains less clear.
2. The burying beetle *Nicrophorus vespilloides* is an insect that exhibits biparental care, relying on small vertebrate carcasses to breed on. Such carcasses are highly valuable but relatively rare in nature, and so are competitively sought out by many other species, including the bluebottle fly, *Calliphora vomitoria*. Despite the close association and frequent rivalry between the two species, how their interactions shape biparental care decisions in these beetles has received little attention.
3. We investigated how perceived risk of interspecific competition affected breeding success and duration of male and female care by staging encounters between breeding pairs of beetles and fly intruders. We presented breeding pairs with dead flies, at varying times and densities, to assess changes in duration of care and fitness outcomes. We used dead flies to ensure the beetles' response was driven solely by cues associated with flies, rather than direct interaction or resource depletion.
4. We found that the duration of both male and female care was plastic in response to the presence of competitors, as both increased their duration of care. However, these encounters also increased breeding failures, with the strongest effects occurring when encounters took place early in the breeding attempt. Our results demonstrate plasticity in parents' behaviour in response to a perceived competitive threat, but this plastic response was associated with increased breeding failures, suggesting a trade‐off between dealing with competitors and completing other parental tasks.

## Graphical abstract

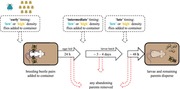

## 1 INTRODUCTION

Parents often care for their offspring to buffer the otherwise detrimental effects of environmental stressors on the fitness of their offspring (Clutton‐Brock, 1991; Royle et al., 2012). In some species, male and female parents cooperate to care for their shared young (Lessells & McNamara, 2011). Known as biparental care, this form of cooperation is favoured when two parents can increase the fitness of their offspring beyond what a single parent could achieve, and when each parent gains a benefit from assisting its partner that outweighs its costs in terms of reduced opportunities to remate with a different partner should it desert the young (Maynard Smith, 1977). Biparental care is often plastic, whereby each parent adjusts its contribution towards care based on its own condition, its partner's contribution or condition (Pilakouta et al., 2015), as well as environmental conditions, such as the presence of predators and food availability (Royle et al., 2014). Such plasticity is essential as it allows each parent to alter its level of care to match the specific circumstances that determine its benefits and costs of care. Furthermore, such plasticity provides some degree of redundancy should one parent die or be injured. For instance, in long‐lived monogamous Cape gannets (*Morus capensis*), when the flying ability of one parent is impaired, its partner compensates with greater nest attendance and increased foraging frequency (Bijleveld & Mullers, 2009). In doing so, breeding success and offspring performance suffer less, as the would‐be cost of reduced allocation from one parent is partially offset by the actions of the other. Similarly, in eastern bluebirds (*Sialia sialis*), the benefits of coordinated parental food provisioning, detected as improved nestling growth, are greater when there is increased intensity in interspecific competition with tree swallows (*Tachycineta bicolor*) (Burdick & Siefferman, 2020). Thus, biparental care may serve as an important buffer against a wide range of unpredictable environmental stressors, including competition for resources (Vincze et al., 2017).

Competition is an important environmental stressor that shapes community structure and drives natural selection through both exploitation and interference (Abrams, 1990; Volterra, 1926). In response, individuals are subject to selective pressures to adopt tactics that enhance their fitness, either by directly outcompeting rivals or by modifying their behaviour to minimise competition, thereby increasing survival and reproductive success (Emlen, 2008; Finke & Snyder, 2008). While some traits that confer competitive advantages—such as body size or weaponry—may be relatively fixed once developed, behavioural responses to competition can be flexible (Snell‐Rood, 2013). Rather than always allocating resources to traits that enhance competitive ability, individuals may instead adopt tactics that allow them to adjust their behaviour dynamically based on their competitive environment (Nussey et al., 2007). For example, *Formica* spp. may shift between aggression or tolerance depending on their assessment of a competitor's competitive ability and the value of the resource under contest (Tanner & Adler, 2009). For interspecific competition, where conflict arises between members of different species, selection may favour cooperation among individuals within a social group, particularly breeding partners, as a means of overcoming increased competitive pressures (Oliveira & Bshary, 2021). Cooperation can also emerge under intraspecific competition, although interspecific interactions may uniquely shape plasticity in biparental care by imposing distinct pressures that require parents to coordinate their efforts in new ways. For instance, breeding partners may need to prioritise joint defence of offspring or adjust their division of labour in response to intruding competitors (Ratz et al., 2022; Zimmermann et al., 2021).

There is good evidence that competition shapes reproductive performance in species with biparental care, such as for intra‐ and interspecific competition in *Parus* spp. (Dhondt, 1977, 2010). Prior research has explored the impact of competitive interactions with microbes (Arce et al., 2012; Biedermann & Rohlfs, 2017; Cotter & Kilner, 2010; Körner et al., 2023; Meunier, 2015; Rozen et al., 2008; Trumbo et al., 2021), or the general effect of competition on the evolution of cooperation (Costa, 2006; Trumbo, 1994; Trumbo & Fiore, 1994; Wilson, 1975). However, there is still little information on how interspecific competition influences the plasticity of male and female care in such species. Such information would help advance our understanding of the effects of interspecific competition in species with biparental care, given that the two parents must somehow coordinate their response to interspecific competition. Indeed, each parent is expected to adjust its contribution towards care in response to the threat of interspecific competition, as well as to its partner's contribution. For example, should one parent respond to greater interspecific competition by increasing its level of care, this may be matched by an increase in care by its partner if the two parents respond in a similar way to this threat. Moreover, parents may be particularly sensitive to certain attributes of competitive encounters—such as the timing and density of encounters (see below for further detail)—which are likely to vary across breeding attempts and could critically shape when and how parents alter their care tactics to maximise reproductive success and resolve conflict (Carlisle, 1982; Sharp & Clutton‐Brock, 2011; Sillett et al., 2004; Wiebe, 2003).

The burying beetle *Nicrophorus vespilloides* provides an excellent system for studying the effects of interspecific competition on the plasticity of biparental care, for the following reasons. Firstly, this species provides extensive biparental care, with both parents contributing to carcass preparation, food provisioning and defence of the brood (Eggert et al., 1998; Smiseth & Moore, 2004), and previous work has demonstrated that social behaviours in *Nicrophorus* spp. are sensitive to the presence of competitors (Trumbo, 1990). *Nicrophorus vespilloides* is known to respond strongly to both intra‐ and interspecific competition with other members of the genus *Nicrophorus* (Bartlett & Ashworth, 1988; Otronen, 1988; Ratz et al., 2022). Secondly, *N. vespilloides* depends on small vertebrate carcasses as a resource for breeding, a rich and ephemeral resource that is contested by a wide range of potential competitors, including conspecifics, other *Nicrophorus* spp., microbial decomposers, opportunistic vertebrate scavengers and many carrion‐breeding insects (Potticary et al., 2024; Scott, 1998; Wilson, 1975). These different competitors vary in their tactics for exploiting carrion, necessitating *Nicrophorus* spp. to employ a wide range of tactics. *Nicrophorus* spp. may engage in direct physical contests with conspecifics for control over carcasses, whereas microbial decomposers rapidly degrade the resource through colonisation, favouring tactics such as resource preservation and rapid breeding (Duarte et al., 2018; Jacobs et al., 2014; Janzen, 1977). Meanwhile, vertebrate scavengers may pose an additional challenge by consuming or removing the carcass entirely, favouring tactics such as concealment. Previous work has demonstrated that *N. vespilloides* and other species in the genus *Nicrophorus* adjust their social behaviours in response to competitors, focusing on the immediate outcomes of competitive interactions, including outcomes of contests over carcasses, responses to conspecific intruders and the effects of body size on fighting ability and reproductive success (Bartlett & Ashworth, 1988; Otronen, 1988; Ratz et al., 2022; Trumbo, 1990). There is now a need for studies investigating how parents subsequently adjust their contribution towards care in response to such interspecific competition.

Here we focus on interspecific competition between *N. vespilloides* and the blowfly *Calliphora vomitoria*. The latter pose a competitive threat to *N. vespilloides* by laying their eggs on carrion, which in turn can lead to an influx of fly larvae that consume the resource and outcompete beetle larvae (Reibe & Madea, 2010). This mode of competition creates a severe constraint on reproductive success, as *N. vespilloides* must either prevent oviposition by flies or respond to the effects of potential competition from maggots once the carcass is colonised. Prior studies across the genus have shown the importance of blowfly competition for shaping reproductive tactics in *Nicrophorus* spp. For instance, in *N. vespilloides*, association with phoretic mites can increase the competitive ability against *C. vomitoria* (Sun & Kilner, 2020). Meanwhile, for both *N. tomentosus* and *N. nepalensis*, communal breeding is promoted through interactions with flyblown carcasses and eggs (Chen et al., 2020; Scott, 1994). Other studies have also highlighted the impact of interspecific competition with flies on reproductive success in *Nicrophorus* spp., and such competition has been proposed as a selective pressure contributing to the evolution of biparental cooperation (Satou et al., 2000; Trumbo, 1994; Trumbo & Fiore, 1994). While *Nicrophorus* spp. are known to bury carcasses, a behaviour that helps conceal the carcass from flies and other scavengers (Scott, 1998), competition at the surface before or during burial is common. *Calliphora vomitoria* may oviposit in the brief window before the carcass is fully concealed, thus making timing of their arrival a critical determinant of reproductive success. Our study has therefore been designed to investigate the parental response during this high‐risk window.

We used a 3 × 2 fully factorial design that focused on two main sources of variation of competitive interactions between the two species; that is, the timing of arrival of *C. vomitoria* in the breeding cycle of *N. vespilloides*, and the density of *C. vomitoria* arriving on the carcass. Both species are adept at detecting carrion, and are often among the first to arrive on a fresh carcass. We tested for plasticity of male and female parental care in response to interspecific competition. We initially tested for plasticity in response to the presence or absence of flies and then extended our analysis to examine plasticity in response to variation in the timing of the arrival of *C. vomitoria* during the beetles' breeding cycle, as well as variation in the density of *C. vomitoria*. We expected that parents would perceive the presence of *C. vomitoria* as a competitor for access to their breeding resource, and predicted that this would lead them to increase the duration of their care as a response to that threat. We also predicted that the perceived presence of *C. vomitoria* would reduce breeding success for two distinct reasons. Firstly, parents facing potential competitors may be forced to divert effort towards tasks, such as carcass preparation and defence, leaving less time for direct offspring care. Secondly, parents may adaptively reduce brood sizes in anticipation of lower or less stable resource quality when competitors are present. By varying the timing and density of *C. vomitoria*, we tested whether parental responses to competition were fixed or plastic in response to key aspects of the presence of an interspecific competitor. Specifically, we predicted that the presence of *C. vomitoria* would have the greatest impact early in the breeding cycle, when parents are most invested in securing the vulnerable carcass. The rationale for this is twofold: first, the earlier the beetles begin breeding relative to the arrival of *C. vomitoria*, the greater the competitive advantage of the beetles, as they would have already secured and prepared the carcass for their offspring. Second, over time, the value of the carcass declines as it is consumed, and larvae become more independent as they approach pupation. Thus, the effect of competition should be greatest early on in the breeding attempt. Similarly, we predicted that a higher density of *C. vomitoria* would have a stronger negative effect on breeding success and a stronger positive effect on the duration of care, as the greater number of *C. vomitoria* would represent a higher level of interspecific competition. Our analysis was exploratory, rather than hypothesis‐driven, with respect to any possible interactions between timing and density of competition.

## 2 MATERIALS AND METHODS

### 2.1 Study system

We used eighth generation beetles from an outbred laboratory population maintained at the University of Edinburgh. All beetles in this population were originally collected in Edinburgh, UK, and were kept at 20°C under a 16:8 h light: dark cycle. All non‐breeding adults were individually housed in clear plastic containers (12 × 8 × 2 cm) lined with moist soil, and fed raw organic beef twice a week. All beetles were non‐mated, between 10 and 30 days old, and had a mass between 0.18 and 0.36 g prior to their use in this experiment. Data available from the Dryad Digital Repository: https://doi.org/10.5061/dryad.6q573n687 (Patmore & Smiseth 2026).

We reared colonies of the carrion‐breeding bluebottle fly, *C. vomitoria* (4 colonies of <500 individuals each), in screened cages (47.5 × 47.5 × 47.5 cm) until pupation under the same lab conditions as described above. We purchased the flies as larvae nearing pupation (larval instar stage 3) from BugzUK. We selected *C. vomitoria* as our candidate competitor due to their prevalence and overlapping distribution with *N. vespilloides* in the UK, and similar dependency on carrion to breed (Charabidze et al., 2021; Matuszewski et al., 2008). Thus, the two species are likely to be frequent rivals for access to carrion in the wild. Previous work has demonstrated that the beetles will react to dead conspecific intruders in a similar way to live ones (Paquet et al., 2017; Ratz et al., 2022; Steiger et al., 2009), and so we chose to use dead, instead of live *C. vomitoria* as a cue for interspecific competition in our design. In doing so, we were able to separate the effects of the presence of *C. vomitoria* on the reproductive decisions of *N. vespilloides* from the potential effects of resource depletion or interference arising through interspecific competition, which would have been confounded had we used live flies. After emerging as adults, *C. vomitoria* were supplied with dry granulated sugar and water ad libitum, along with twice daily feedings of chicken liver to promote the maturation of their ovaries (Sivell et al., 2023). One week later, when all *C. vomitoria* in the colony were sexually mature, they were euthanised by being frozen at −20°C, and stored in batches of <20 individuals in 50 mL centrifuge tubes for later use. No *C. vomitoria* that had been stored for longer than 2 weeks were included in this experiment, to ensure that any chemical cues from the cuticular hydrocarbons of the *C. vomitoria* were preserved as much as possible (Singer, 1998; Steiger et al., 2009). All *C. vomitoria* were thawed for 10 min immediately before use.

To ensure that our study focused solely on parental care decisions in response to interspecific competition, we removed mites from all beetles before the experiment. Mites are known to influence competitive dynamics by consuming fly eggs, which could in turn alter the observed effects of competition (Nagel et al., 2025; Springett, 1968). By eliminating mites as a confounding variable, we ensured that any changes in beetle reproductive behaviour could be attributed specifically to the presence of *C. vomitoria* rather than indirect effects arising through the presence of mites (Nagel et al., 2025; Sun & Kilner, 2020).

### 2.2 Experimental design

We used a 3 × 2 factorial design to test how variation in the perceived risk of competition with *C. vomitoria* influences parental care plasticity in *N. vespilloides*. We manipulated both the timing of encounters (‘early’, ‘intermediate’ and ‘late’) and the density of *C. vomitoria* being encountered (‘low’ and ‘high’). Timing levels were defined as follows: ‘early’ when flies were added immediately ahead of the time of parental pairing (i.e. 0 h after we placed the male and female on the carcass), corresponding to a period when parents should be most sensitive to disruption; ‘intermediate’ when flies were added 24 h after pairing, corresponding to a period when egg‐laying is underway but before larval hatching; and ‘late’ when flies were added shortly after the first larvae began hatching (typically 72 h after pairing), corresponding to a period when parents face minimal competitive risk but the carcass remains valuable. In all cases, *C. vomitoria* were placed directly on the carcass. Density levels were defined as follows: ‘low’ when only one *C. vomitoria* was present; and ‘high’ when 10 *C. vomitoria* were present, effectively surrounding the carcass. These factors were chosen because arrival time and density of *C. vomitoria* vary naturally and are expected to influence the competitive dynamics between the two species at different stages of the breeding attempt (Charabidze et al., 2021; Matuszewski et al., 2008; Scott, 1998). To provide a baseline reference, we also included a control treatment where no *C. vomitoria* were present at any stage. All treatment groups were replicated 36 times, yielding a total sample size of *N* = 252. All treatment groups, including the control, were tested in smaller batches over 18 weeks, and each batch contained all treatment types.

**FIGURE 1:**
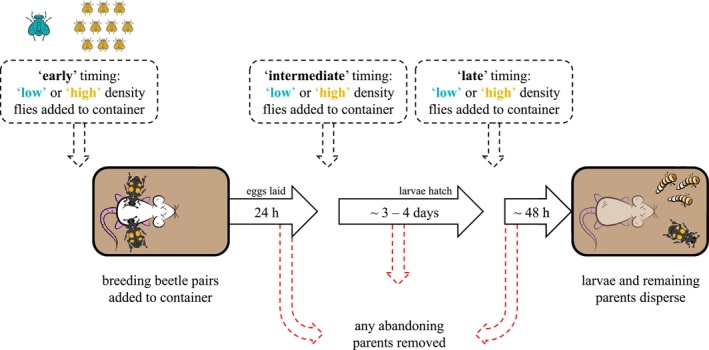
Schematic for the experimental design and procedures. Breeding pairs of *N. vespilloides* were added to a container with a fresh mouse, to breed, rear their larvae and disperse as indicated by solid black arrows. Flies were added to containers according to two protocols: (1) Treatments for timing were added at only one point as appropriate (black dashed boxes: ‘early’, before parent pairs; ‘intermediate’, after 24 h when eggs had been laid; or ‘late’, immediately after larvae hatched.) (2) Treatments for density were either low (blue, 1 fly) or high (orange, 10 flies). Red dotted arrows indicate that abandoning parents, defined as those missing from the crypt after two consecutive checks, would be removed immediately.

We prepared large plastic containers (17 × 12 × 6 cm) lined with a thin layer (~1 cm) of moist soil. We added a previously frozen large (17–22 g, average ± SE = 19.766 ± 0.082 g) mouse carcass (Livefoods Direct, Sheffield, UK) to each chamber, to control for variation in carcass mass (Smiseth & Moore, 2002). Next, we randomly paired unrelated male and female beetles, assigned them to one of our treatment groups, weighed them both and placed each pair into their respective prepared containers together, as per our experimental design (Figure 1). Following this, we assessed the parents' initial behavioural responses to the dead *C. vomitoria*, observed immediately, as well as after 24 h by scoring visible damage to the flies (damaged, missing or intact) as well as position (within crypt, or removed outside of crypt), which allowed us to monitor how parents were detecting and responding to interspecific competitors. We did not replace or remove any flies from any chamber until 24 h had passed, so as to avoid repeated disturbances to the breeding chambers and minimise any additional sources of interference to the beetles.

We inspected the containers for parental abandonment twice daily (10:00 AM and 06:00 PM) from the moment the male and female were first paired with the carcass in the breeding chamber until the time of dispersal. This moment marks the start of the breeding attempt for all measurements. We scored parents as having abandoned the brood if they were neither on the carcass nor the crypt (i.e. the depression in the soil surrounding the carcass) for two consecutive checks, according to protocol previously established for this species (Pilakouta et al., 2018; Ratz et al., 2021). In the case of any parental abandonment, we immediately removed the individual in question, to avoid risk of infanticide, and recorded the parents' mass change from pairing until dispersal (Bartlett, 1987; Richardson & Smiseth, 2021). Duration of care was recorded as the number of hours elapsed from the moment of pairing until abandonment, or until larvae had dispersed if that parent remained with their brood until this time. At the time of dispersal, which is when larvae will independently move away from the carcass, we recorded the total number of larvae in the brood and calculated the average larval mass for the brood to the nearest 0.1 mg on a digital weighing balance (Ohaus™ Pioneer).

### 2.3 Statistical analysis

All analyses were carried out using R version 4.3.2 (R Core Team, 2013), using packages ‘car’ (Fox et al., 2001), ‘MASS’ (Ripley et al., 2017), and ‘DHARMa’ (Hartig & Hartig, 2017) to assist with our model selection and diagnostics.

Our analysis was carried out in three stages. Firstly, we assessed whether beetle parents were responding to the presence of *C. vomitoria* to confirm that our treatments had the intended effect. This was done firstly through observations of negative parental interactions with *C. vomitoria* shortly after their introduction, which included ‘charge‐like’ behaviours where the beetles would suddenly rush forward to engage with, and quickly release, the dead flies. We also recorded instances of wing, leg and head removal—hereafter referred to as ‘damaged’—as well as cases where beetles would carry fly remains away from carcasses to the outer edge of the breeding chamber—referred to as ‘removed’. To validate and quantify these observations, we recorded how often *C. vomitoria* were damaged or removed from the breeding chambers in each of our experimental treatments after 24 h. Next, we tested for effects of the presence and absence of *C. vomitoria* on breeding success, the duration of male and female care, parental mass change and measures of reproductive fitness: total brood size and average larval mass. For our analyses on duration of care, parental mass change and reproductive fitness, only broods for which larvae hatched were included (*N* = 176). For this, we compared our control treatment, where flies were absent, against all other treatment groups, where flies were present. We pooled all fly‐present treatments and used a non‐parametric Mann–Whitney *U*‐test—to account for skew between—for both male and female duration of care. We used a Fisher's exact test to compare breeding success, and all remaining fitness measures listed above.

Finally, we carried out analysis on our 3 × 2 factorial design to test for the effect of the timing of encounters with *C. vomitoria* and the density of *C. vomitoria*. We used a logistic regression model for breeding success (*N* = 216). We used linear regression models for normally distributed responses, such as male and female duration of care, parent mass changes, larval brood size and mass at dispersal. As before, only broods for which larvae hatched were included in these analyses (*N* = 142). In all models, we initially included the timing of encounters and the density of *C. vomitoria* as fixed effects. We initially fitted models, including the interaction between timing and density. Model support was evaluated using Akaike information criterion (AIC), and where interaction terms were unsupported (AIC <2 relative to the additive model; Table S1) and not statistically significant, we present the simpler additive models (Table 1). The interaction was retained only for the larval brood size model, where it improved model support. For parent mass changes, as well as larval mass, we also included larval brood size as an additional fixed effect to compensate for any effect variation in natural brood sizes may have had on parental care or larval growth. To further interpret the main effect of timing across all our analyses, we carried out Tukey‐adjusted pairwise comparisons using estimated marginal means, from the package ‘emmeans’ (Lenth & Piaskowski, 2025). Full pairwise results are presented in Tables S2 and S3.

**TABLE 1:** Summary of statistical tests for the effects of *C. vomitoria*, at different times (early, intermediate and late) and density (low or high), on breeding success, and both male and female duration of care.

Response	Predictors	Odds ratios/estimates	95% CI	*p*
Breeding success	(Intercept)	1.38	0.80 to 2.41	0.249
	Intermediate	1.34	0.69 to 2.63	0.395
	Late	2.45	1.21 to 5.09	**0.014**
	High	0.92	0.52 to 1.63	0.771
Male duration	(Intercept)	107.46	100.93 to 113.99	**<0.001**
	Intermediate	−2.23	−10.39 to 5.93	0.589
	Late	−13.74	−21.54 to −5.94	**0.001**
	High	−0.33	−6.71 to 6.05	0.919
Female duration	(Intercept)	119.14	114.97 to 123.30	**<0.001**
	Intermediate	4.47	−0.73 to 9.67	0.091
	Late	−0.12	−5.09 to 4.85	0.962
	High	−1.93	−5.99 to 2.14	0.350

*Note*: Estimates are shown with 95% confidence intervals (CI). Reference category was for the interaction between timing and density (early × low). Statistically significant *p* values (<0.05) are shown in bold. *N* = 216.

## 3 RESULTS

### 3.1 Damage to *C. vomitoria*

We first tested for physical damage to *C. vomitoria* to verify that our treatments had the intended effect of simulating the presence of competitors. As intended, parents reacted strongly to the presence of dead *C. vomitoria* by either damaging or removing them. We often observed parents charging towards the dead *C. vomitoria* in a similar manner to how they charge towards conspecific intruders. We found damage to 883 out of the 1123 *C. vomitoria* used in this study (~79% of all cases). Of the remaining 240 that were undamaged after 24 h, 183 had been removed from the crypt (~76% of undamaged flies or ~16% of all cases). As expected, a greater proportion of *C. vomitoria* were damaged or removed in our early treatments compared with the late treatments (*χ* ^2^ = 42.081, *p* = 0.001).

### 3.2 Plasticity in response to presence or absence of competitors

**FIGURE 2:**
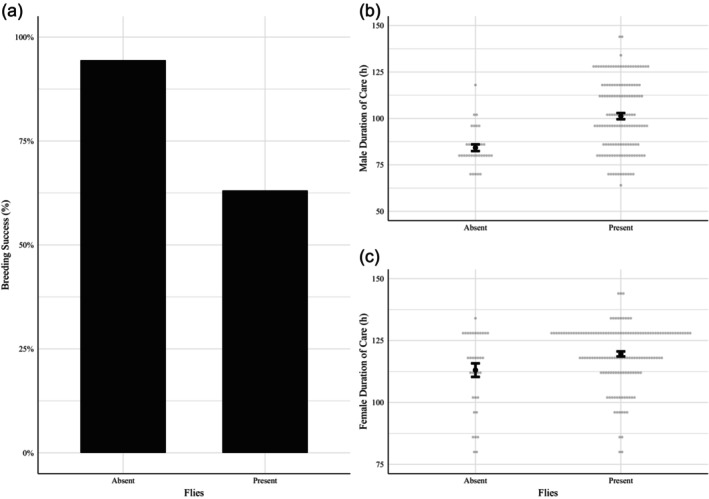
Parental responses to the absence or presence of *C. vomitoria*, showing: (a) Breeding success, as a % of all broods that failed to produce surviving larvae at dispersal. (b) Point estimates (mean ± SE) for male duration of care, as the time in hours males remained with their brood. (c) Point estimates (mean ± SE) for female duration of care, as the time in hours females remained with their brood. For (b and c), black points represent means, while grey points indicate individuals.

The presence of *C. vomitoria* had a negative effect on breeding success (Figure 2a). In our 36 control replicates where *C. vomitoria* were absent, only two pairs failed to produce and rear offspring to dispersal (~5% failure), compared with 74 out of the 216 replicates for the pooled treatments where *C. vomitoria* were present (~34% failure, Fisher's Exact, *p* < 0.001).

Parents responded plastically to the presence of *C. vomitoria*. Both male and female parents remained with their brood to provide care for longer when *C. vomitoria* were present (male duration of care ± SE = 101 ± 1.67 h; female duration of care ± SE = 120 ± 2.74 h) than when absent (male duration of care ± SE = 84.2 ± 1.79 h, *U* = 1179.5, *p* < 0.001; female duration of care ± SE = 113 ± 1.03 h, *U* = 1868.5, *p* = 0.034; Figure 2b,c). There was no evidence that the presence or absence of *C. vomitoria* affected male mass change (*C. vomitoria* present ± SE = −0.005 ± 0.002 g; *C. vomitoria* absent ± SE = −0.003 ± 0.004 g, *U* = 2484, *p* = 0.795), female mass change (*C. vomitoria* present ± SE = −0.024 ± 0.003 g; *C. vomitoria* absent ± SE = −0.016 ± 0.005 g, *U* = 2832, *p* = 0.118), number of larvae surviving until dispersal (*C. vomitoria* present ± SE = 13 ± 0.71; *C. vomitoria* absent ± SE = 14.8 ± 1.04, *U* = 2872, *p* = 0.087) or average larval mass at dispersal (*C. vomitoria* present ± SE = 0.198 ± 0.003; *C. vomitoria* absent ± SE = 0.197 ± 0.007, *U* = 2383, *p* = 0.909).

### 3.3 Plasticity in response to variation in timing of encounters and density

**FIGURE 3:**
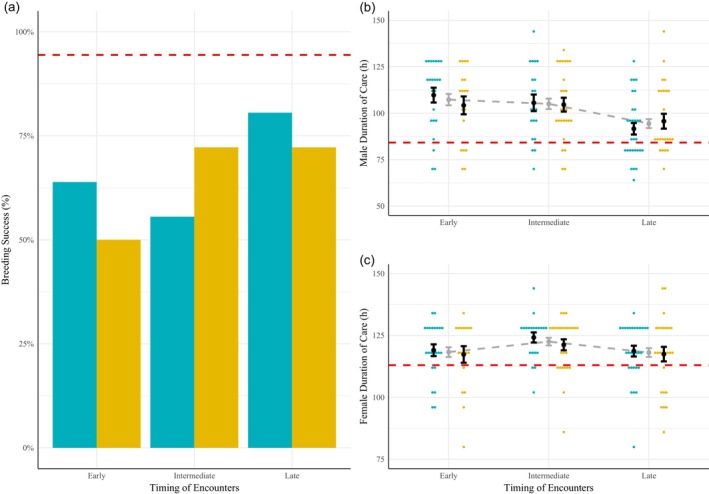
Parental responses to the timing of encounters with *C. vomitoria* and density of *C. vomitoria* (according to our 3 × 2 design). *X*‐axis represents our treatment for timing (left–right: early, intermediate, late), while colour represents density (blue: low, 1 fly; orange: High, 10 flies) showing: (a) Breeding success, as a % of all broods that failed to produce surviving larvae at dispersal. (b) Point estimates (mean ± SE) for male duration of care, as the time in hours males remained with their brood; (c) Point estimates (mean ± SE) for female duration of care, as the time in hours females remained with their brood. For (b and c), black point represents mean, colours indicate individuals, while grey indicates the overall mean for that timing treatment. The red dashed line indicates the control fly‐absent value, extended from Figure 2, for reference.

Focusing on the six treatments in our factorial design where *C. vomitoria* were present, we found some evidence for plasticity in the parents' responses to the timing of encounters with *C. vomitoria*. The timing of encounters had an effect on the duration of male care provided, with male care found to be longest when *C. vomitoria* were encountered earlier in the breeding attempt (male duration of care ± SE: early = 107 ± 3.06 h; intermediate = 105 ± 2.83 h; late = 93.6 ± 2.50 h; Table 1, Figure 3b). In contrast, the timing of encounters with *C. vomitoria* had no effect on the duration of female care (female duration of care ± SE: early = 118 ± 1.96 h; intermediate = 123 ± 1.54 h; late = 118 ± 1.78 h; Table 1, Figure 3c). Breeding success was lowest when *C. vomitoria* were encountered early (~35% failure) rather than late (~16% failure; Table 1; Figure 3a). We found no evidence that the density of *C. vomitoria* had an effect on breeding success or duration of care for either parent, nor for the interaction between timing and density (Figure 3a–c).

The timing of the arrival of *C. vomitoria* had an effect on brood size at the time of larval dispersal from the carcass. Parents that encountered *C. vomitoria* earlier in their breeding attempt had smaller brood sizes (average ± SE = 8.32 ± 1.08) than those that encountered *C. vomitoria* at a later stage (average ± SE = 13.6 ± 1.31; Table 2, Figure S1). There was no evidence that the timing of the arrival of encounters with *C. vomitoria*, their density or the interaction (where included) between them had an effect on any of our additional fitness outcomes, including male and female mass change during breeding, or the average larval brood mass at dispersal (Table 2).

**TABLE 2:** Summary of statistical tests for the effects of *C. vomitoria*, at different times (early, intermediate and late) and density (low or high), on both male and female mass change, number of larvae at dispersal and average larval mass at dispersal.

Response	Predictors	Estimates	95% CI	*p*
Male mass change	(Intercept)	−0.01	−0.02 to 0.01	0.314
	Intermediate	−0.00	−0.01 to 0.01	0.753
	Late	−0.01	−0.02 to 0.00	0.210
	High	−0.00	−0.01 to 0.00	0.357
	Number of larvae	0.00	−0.00 to 0.00	0.124
Female mass change	(Intercept)	−0.03	−0.04 to −0.02	**<0.001**
	Intermediate	0.00	−0.01 to 0.02	0.503
	Late	0.01	−0.00 to 0.02	0.182
	High	−0.01	−0.02 to 0.00	0.174
	Number of larvae	0.00	−0.00 to 0.00	0.224
Number of larvae	(Intercept)	9.26	5.98 to 12.54	**<0.001**
	Intermediate	2.34	−2.34 to 7.15	0.338
	Late	9.01	4.62 to 13.41	**<0.001**
	High	0.63	−4.33 to 5.58	0.803
	Intermediate × High	1.20	5.62 to 8.01	0.729
	Late × High	−5.75	−12.28 to 0.78	0.084
Larval mass	(Intercept)	0.20	019 to 0.22	**<0.001**
	Intermediate	0.00	−0.01 to 0.02	0.695
	Late	−0.01	−0.02 to 0.01	0.279
	High	−0.01	−0.02 to 0.00	0.138
	Number Larvae	0.00	−0.00 to 0.00	0.536

*Note*: Estimates are shown with 95% confidence intervals (CI). Reference category was for the interaction between timing and density (early × low). Statistically significant *p* values (<0.05) are shown in bold. *N* = 142.

## 4 DISCUSSION

We found that breeding *N. vespilloides* parents responded strongly to the presence of dead *C. vomitoria*, inflicting damage to and/or removing them from the crypt. This confirmed that our experimental treatments had the intended effect of simulating the presence of competitors and is consistent with previous work on this species showing that breeding parents respond to dead conspecific intruders as if they were alive (Steiger et al., 2009). Indeed, ~95% of our dead *C. vomitoria* were either damaged (79%) or removed from the crypt (16%) compared with ~70% of dead conspecific intruders as reported by Ratz et al. (2022). Thus, the response to the presence of dead *C. vomitoria* was as strong as, if not stronger than, that to dead conspecific intruders. Using dead *C. vomitoria* was critical to our design because it allowed us to attribute changes in parental behaviour and offspring performance to cues of the presence of competitors rather than to resource depletion by *C. vomitoria*. Had we used live *C. vomitoria*, their maggots may have consumed resources, thereby introducing confounding effects on parent‐offspring and male–female interactions mediated through resource depletion. By using dead *C. vomitoria*, we eliminated these confounding effects, thereby ensuring that any observed responses reflected plasticity in parental behaviour alone.

Our first main finding was that the presence or absence of dead *C. vomitoria* had an impact on plasticity in parental care and breeding success. Both male and female parents responded plastically to the presence of dead *C. vomitoria* by increasing their duration of care. This finding supports our prediction that parents would increase their care in response to cues indicating a heightened level of interspecific competition. The rationale for this prediction is that, carrion being a rich and ephemeral resource, parents should increase their allocation to parental care as it serves as a defence mechanism, helping to safeguard the resource and brood from usurpation by competitors. Alternatively, extended care could function as a compensatory tactic, whereby parents allocate more to care for offspring under greater levels of competition to offset any initial fitness costs due to competition. Consequently, parents may prolong care to offset an increased risk of brood loss due to intruder usurpation and use of the carcass. An alternative explanation is that the increased duration of care reflected a delay in the onset of breeding if competitors caused parents to start the onset of egg‐laying later than they otherwise would. However, unpublished data show that female *N. vespilloides* actually accelerate the onset of egg‐laying in the presence of *C. vomitoria* (Patmore 2025). This shows the extended duration of care is not simply a by‐product of delayed reproduction, suggesting that it does reflect plasticity in post‐hatching parental care.

We found that there was increased risk of breeding failure in the presence of dead *C. vomitoria*. As argued above, using dead *C. vomitoria* shows that this decline in breeding success was due to parental responses to cues of the presence of competitors rather than to other factors, such as resource depletion by *C. vomitoria*. The finding that breeding success was lower in the presence of dead *C. vomitoria* is surprising, given our first main finding that both male and female parents increased their duration of care in response to *C. vomitoria*. The reason for this is that an increase in care is typically associated with improved offspring performance and greater breeding success (Webb et al., 2010). Furthermore, prior work shows that duration of care is positively correlated with time spent on direct and indirect care in *N. vespilloides* (Pilakouta et al., 2024). Thus, our results contradict what we assumed would be the likely causal pathway, whereby the presence of *C. vomitoria* triggered an increase in parental care, which in turn should enhance offspring performance and ultimately breeding success. Reduced offspring performance and breeding success is often associated with resource depletion by competitors (Matuszewski et al., 2008), but as outlined earlier, we can exclude this mechanism because our design used dead flies that could not consume carrion. Indeed, had we used live *C. vomitoria*, we might have erroneously attributed reduced breeding success to resource depletion by *C. vomitoria*. As discussed below, our results suggest that the presence of *C. vomitoria* negatively impacted offspring performance through some as yet unknown mechanism, which in turn prompted parents to increase their duration of care in an attempt to compensate for this negative impact.

What mechanisms might reduce offspring performance in the presence of dead *C. vomitoria* despite the increased allocation to parental care? Our study was not designed to identify such mechanisms, but given existing knowledge of the study system, we propose that one such mechanism could involve a shift in parental behaviour combined with sex‐specific parental roles. Biparental care is often associated with sex‐specific parental roles. For example, in convict cichlids (*Archocentrus nigrofasciatus*), females spend more time near the offspring, while males focus more on patrolling the territory and chasing away intruders (Itzkowitz et al., 2001). Meanwhile, in orange‐tufted sunbirds (*Nectarinia osea*), females provision more food to the nestlings, while males allocate more time to mobbing potential predators (Markman et al., 1996). In *N. vespilloides*, females typically engage in direct care, such as food provisioning of larvae, while males focus on indirect care, such as guarding the carcass from competitors (Socias‐Martínez & Kappeler, 2019). Indirect care occurs on the outside of the carcass and may therefore provide a better vantage point for detecting and confronting intruders, while direct care, in contrast, occurs within the crater of the carcass. If parents respond to cues of the presence of *C. vomitoria* as an increased threat to the brood, both parents may shift their efforts from direct care to indirect care. This shift may lead to a subsequent reduction in larval performance and breeding success as larvae would get less pre‐digested food from the female parent. Still, this explanation does not fully account for our observed decline in breeding success, as it would predict the negative effects to manifest after hatching when parents must divide their time between direct and indirect care. Meanwhile, our results suggest that breeding failure occurs at an earlier stage. Thus, parents may respond to cues about the presence of competitors by prioritising the monitoring and deterring of *C. vomitoria* before hatching over important pre‐hatching behaviours, such as allocation of resources and time to egg‐laying and carcass preparation. This increased allocation in detecting and repelling intruders may come at the cost of reproductive activities, leading to a failure in breeding before offspring hatch. If parents perceive *C. vomitoria* as a substantial risk to their current breeding attempt, they may reduce allocation in current reproduction, resulting in lower overall breeding success despite prolonged duration of parental care.

Our second main finding was that the timing of encounters with dead *C. vomitoria* had an impact on the plasticity in the duration of male care and breeding success, while there was no effect of the density of dead *C. vomitoria*. This demonstrates that male care (but not female care) is plastic in response to more subtle variation in the timing of encounters with competitors and not just the presence of competitors. This result was driven by the difference between our early and late treatments, with males providing care for longer when encountering *C. vomitoria* early than when encountering *C. vomitoria* late. This highlights the importance of the timing of encounters with cues of the perceived threat and supports our predictions that parents should be most responsive to competitive encounters during the early stages of breeding, when carcasses are most vulnerable and decisions about resource allocation are more critical (Charabidze et al., 2021; Ibáñez‐Álamo et al., 2015; Robertson, 1993). Studies on other systems also demonstrate greater sensitivity to the timing rather than the quantity of cues about potential threats. For example, early exposure to predators alters parental food provisioning in birds (Ibáñez‐Álamo et al., 2015), tadpoles learn and adjust antipredator behaviour based on temporal context of predator cues (Mathis et al., 2008), and songbirds modify reproductive investment when exposed to predation risk cues during breeding (Zanette et al., 2011). In our study, we found that brood size was lowest when *C. vomitoria* were encountered early. This finding may suggest that parents perceive the resource as being of lower quality or under more substantial threat when encountering *C. vomitoria* early. If so, parents might adaptively reduce their clutch size or increase filial cannibalism to produce a smaller, but more viable, brood that matches the perceived reduced value of the resource. Keeping in mind that this was our only treatment group where *C. vomitoria* were present prior to the start of egg‐laying, this result may suggest that parents prioritise detecting and repelling intruders as discussed above.

There was no support for the prediction that a higher density of *C. vomitoria* would have a stronger negative effect on breeding success and a stronger positive effect on the duration of care, as the greater number of competitors would represents more intense competition with *C. vomitoria*. This finding suggests that breeding parents are sensitive to cues of the mere presence of *C. vomitoria* and the timing of such cues but not to cues of the density of *C. vomitoria*. Thus, it may be more important for breeding parents to respond to cues of the presence or absence of competitors, and the timing of their presence, than the density of competitors. This might make sense in terms of what we know about the natural history of *N. vespilloides* and *C. vomitoria*. Both species are specialised for breeding on carrion, and are among the first species to arrive at a carcass (Charabidze et al., 2021; Potticary et al., 2024). In the wild, *N. vespilloides* will attempt to bury carcasses promptly and apply antimicrobial secretions, which specifically serve to reduce the risk of both intra‐ and interspecific competition by concealing the carcass (Suzuki, 2000; Trumbo et al., 2021). Our experimental setup did not allow the breeding beetles to fully bury the carcass, thereby allowing for continued surface‐level encounters throughout breeding. As such, our experimental setup represents a scenario where beetles have not successfully buried the resource. This might happen in the wild as *N. vespilloides* occasionally will bury their carcass very shallowly in the leaf litter if the substrate prevents them burying it deeper in the soil. Nevertheless, the frequency of encounters with *C. vomitoria* after hatching, as assessed in our design, will inevitably be lower in natural situations.

It is important to acknowledge limitations to our cue‐based experimental design. It is possible that parents soon detected that the dead *C. vomitoria* were not a real competitive threat to their breeding attempt. Nevertheless, the charge‐like behaviours we observed confirmed that parents showed a strong initial response to the cue prompted by their presence and our results for the duration of care discussed above show that this cue was sufficient to trigger plastic changes in male parental care. That said, it is likely that the use of live, interacting competitors would have elicited an even stronger or more complex response. Studies using live flies or flyblown carcasses have shown that actual interspecific competition can promote cooperation, including biparental care and communal breeding, because removing eggs and larvae requires a coordinated effort (Chen et al., 2020; Scott, 1994; Trumbo & Fiore, 1994). Our study complements these earlier findings by demonstrating that the parental response to the threat of competition is separate from any responses to the effects of resource depletion. The presence of dead *C. vomitoria* alone was enough to increase the duration of care provided by male and female parents, and also led to a decrease in breeding success. As discussed in greater detail above, these findings suggest that parents may have shifted their efforts to alternative tactics to negate the perceived effects of competition at the expense of other reproductive tasks during the early stages of breeding, exposing a trade‐off that may be less evident when there also is resource depletion. We encourage future work in this field to compare responses towards dead and live competitors.

Our study contributes to our understanding of the effects of interspecific competition in species with biparental care where male and female parents must somehow coordinate their response to interspecific competition. This is a key gap in our knowledge as we would expect each parent to adjust its contribution towards care to the treat of interspecific competition, as well as to their partner's contribution. Prior work on species with biparental care highlights the key role played by sexual conflict between caring parents in shaping the behavioural rules determining how each parent responds to a change in the level of care provided by its partner (Lessells, 2012). For example, there is good empirical evidence that coordination of care between male and female parents in passerine birds is based on negotiation; that is, when one parent reduces its contribution towards care, the partner responds by increasing its contribution but not to the extent that it fully compensates for the reduction in care by the other parent (Harrison et al., 2009; Wright & Cuthill, 1989). These behavioural rules must somehow play a role also when the two parents respond to environmental stressors, such as interspecific competition. Our study provides some evidence that the two parents coordinate their responses by increasing their duration of care in response to cues of the presence of *C. vomitoria*, while only male parents respond by increasing their duration of care in response to the timing of such cues. There is now a need for more work in this field. For example, we measured only one aspect of parental care: the duration of care by males and females. Parental care in *N. vespilloides* involves multiple behaviours expressed before and after hatching, including indirect care, such as maintenance of the carcass and guarding of the brood, and direct care, such as food provisioning (Andrews et al., 2017; Walling et al., 2008). Likewise, parental care in passerine birds also involves multiple behaviours expressed before and after hatching, including nest building, incubation, food provisioning, nest sanitation and defence against predators (e.g., Skutch, 1976). Thus, there is now a need for studies that monitor the effects of interspecific competition on the amount of time that male and female parents spend on different parental behaviours, ideally both before and after hatching. Such studies would help understand whether the presence of *C. vomitoria* shifted parental effort towards behaviours that would allow the monitoring and deterring of *C. vomitoria* before hatching over important pre‐hatching behaviours and/or whether the presence of *C. vomitoria* shifted parental effort towards indirect care such as carcass maintenance at the expense of direct care such as food provisioning of larvae after hatching. Furthermore, such studies would help us understand whether parental responses to environmental stressors are shaped by parental rules that evolved in the context of sexual conflict over care. For example, in *N. vespilloides*, such rules are detected as negative correlations between time spent on indirect or direct care by male and female parents (Smiseth & Moore, 2004). One possible avenue for further work that we encourage is to compare these negative correlations in the presence and absence of *C. vomitoria*.

In summary, we present evidence of plasticity in parental care for the burying beetle *N. vespilloides* in response to cues indicating an increased risk of interspecific competition from the blowfly *C. vomitoria*. Our results provide some evidence for coordination between male and female parents in their responses to interspecific competition in a species with biparental care. There is growing evidence that environmental stressors, including competition, predation, parasites and pathogens, and climate change, are key drivers of behavioural plasticity in biparental care or other forms of sociality. For example, ecological stressors influence collective defence and reproductive allocation in social insects, such as *Reticulitermes flavipes* (Tian et al., 2017), as well as cooperative investment and group stability in sociable weavers (*Philetairus socius*) (Fortuna et al., 2024). Our study adds to this work by showing that the effects of cues about the presence of interspecific competitors can be complex and counter‐intuitive; that is, such cues may have a negative impact on breeding success even though male and female parents increased their time spent on parental care.

## AUTHOR CONTRIBUTIONS

Casey Patmore and Per T. Smiseth conceived the ideas and designed the methodology. Casey Patmore collected and analysed the data, and led the writing of the manuscript. All authors contributed critically to the drafts and gave final approval for publication.

## CONFLICT OF INTEREST STATEMENT

None to declare.

## Supporting information


**Table S1.** Model comparisons: AIC (Akaike information criterion) and AICc (Corrected AIC). Values in bold indicate a difference of <2, and thus the preferred model. For any comparisons without bolding, the Additive model—being simpler—was favoured.
**Table S2.** Estimated marginal means (emmeans) comparisons for Table 1. Pairwise comparisons are shown for the effects of timing of encounter with *C. vomitoria* (Early, Intermediate, and Late) on breeding success, and male and female duration of care. Statistically significant differences (*p* < 0.05) are shown in bold.
**Table S3.** Estimated marginal means (emmeans) comparisons for Table 2. Pairwise comparisons are shown for the effects of timing of encounter with *C. vomitoria* (Early, Intermediate, Late) on both male and female mass change, number of larvae at dispersal and average larval mass at dispersal. Statistically significant differences (*p* < 0.05) are shown in bold.
**Figure S1.** Larval brood size in response to the absence or presence of *C. vomitoria* (A) and to the timing and density of *C. vomitoria* (according to our 3 × 2 design: B). A. Comparison between fly‐absent and fly‐present treatments, showing point estimates (mean ± SE) for larval brood size; black points represent treatment means, while grey points indicate individuals. B. Larval brood size across timing (left—right: early, intermediate, late) and fly density (blue: low, 1 fly; orange: high, 10 flies), showing point estimates (mean ± SE); black points represent treatment means, coloured points indicate individuals, while grey indicates the overall mean for that timing treatment. The red dashed line indicates the control fly‐absent value, extended from panel A, for reference.

## Data Availability

Data will be uploaded and made available at the Dryad Digital Repository shortly—https://doi.org10.5061/dryad.6q573n687.
