# Supplementary material for: Plasticity in parental care: Interspecific competitor cues shape biparental cooperation in a burying beetle
Source: J Anim Ecol. 2026 May 4;95(8):1358–71. doi: 10.1111/1365-2656.70251 (PMC13439586; doi:10.1111/1365-2656.70251)
Supplement: Supplementary file 1 [file JANE-95-1358-s001.docx]

***Table S1.*** *Model comparisons: AIC (Aikake Information Criterion) and AICc (Corrected AIC). Values in bold indicate a difference of <2, and thus the preferred model. For any comparisons without bolding, the Additive model – being simpler – was favoured.*

| *Model Type* | *df* | *AIC* | *AICc* |
| --- | --- | --- | --- |
| **Breeding Success:** |  |  |  |
| Interactive | 6 | 279.0085 | 279.4104 |
| Additive | 4 | 279.2231 | 279.4126 |
| **Male Duration:** |  |  |  |
| Interactive | 7 | 1249.3486 | 1250.184 |
| Additive | 5 | **1246.8715** | **1247.313** |
| **Female Duration:** |  |  |  |
| Interactive | 7 | 1122.7853 | 1123.621 |
| Additive | 5 | **1118.9203** | **1119.361** |
| **Male Mass Change:** |  |  |  |
| Interactive | 8 | -619.4625 | -618.3798 |
| Additive | 6 | **-623.4572** | **-622.835** |
| **Female Mass Change:** |  |  |  |
| Interactive | 8 | -581.5661 | -580.4834 |
| Additive | 6 | **-584.1307** | **-583.5084** |
| **Number Larvae:** |  |  |  |
| Interactive | 7 | **1000.1401** | **1000.976** |
| Additive | 5 | 1002.7542 | 1003.195 |
| **Larval Mass:** |  |  |  |
| Interactive | 8 | -525.1812 | -524.0985 |
| Additive | 6 | **-527.9165** | **-527.2934** |

***Table S2.*** *Estimated marginal means (emmeans) comparisons for Table 1. Pairwise comparisons are shown for the effects of timing of encounter with C. vomitoria (Early, Intermediate, Late) on breeding success, and male and female duration of care. Statistically significant differences (P < 0.05) are shown in bold.*

| *Response* | *Estimate* | *SE* | *t / z Ratio* | *P* |
| --- | --- | --- | --- | --- |
| **Breeding Success:** |  |  |  |  |
| Early – Intermediate | -0.291 | 0.342 | -0.851 | 0.671 |
| Early – Late | -0.895 | 0.366 | -2.447 | **0.038** |
| Intermediate – Late | -0.604 | 0.370 | -1.630 | 0.233 |
| **Male Duration:** |  |  |  |  |
| Early – Intermediate | 2.23 | 4.13 | 0.541 | 0.851 |
| Early – Late | 13.74 | 3.95 | 3.483 | **0.002** |
| Intermediate – Late | 11.51 | 3.83 | 3.004 | **0.009** |
| **Female Duration:** |  |  |  |  |
| Early – Intermediate | -4.472 | 2.63 | -1.701 | 0.209 |
| Early – Late | 0.119 | 2.51 | 0.047 | 0.999 |
| Intermediate – Late | 4.591 | 2.44 | 1.880 | 0.148 |

***Table S3.*** *Estimated marginal means (emmeans) comparisons for Table 2. Pairwise comparisons are shown for the effects of timing of encounter with C. vomitoria (Early, Intermediate, Late) on both male and female mass change, number of larvae at dispersal, and average larval mass at dispersal. Statistically significant differences (P < 0.05) are shown in bold.*

| *Response* | *Estimate* | *SE* | *t / z Ratio* | *P* |
| --- | --- | --- | --- | --- |
| **Male Mass Change:** |  |  |  |  |
| Early – Intermediate | 0.00181 | 0.00575 | 0.315 | 0.945 |
| Early – Late | 0.00719 | 0.00571 | 1.260 | 0.420 |
| Intermediate – Late | 0.00538 | 0.00534 | 1.008 | 0.573 |
| **Female Mass Change:** |  |  |  |  |
| Early – Intermediate | -0.00441 | 0.00660 | -0.669 | 0.782 |
| Early – Late | -0.00874 | 0.00655 | -1.334 | 0.379 |
| Intermediate – Late | -0.00433 | 0.00613 | -0.706 | 0.760 |
| **Number Larvae:** |  |  |  |  |
| *Low: |  |  |  |  |
| Early – Intermediate | -2.339 | 2.44 | -0.961 | 0.603 |
| Early – Late | -9.015 | 2.22 | -4.054 | **0.000** |
| Intermediate – Late | -6.676 | 2.31 | -2.884 | **0.013** |
| *High: |  |  |  |  |
| Early – Intermediate | -3.534 | 2.44 | -1.447 | 0.320 |
| Early – Late | -3.265 | 2.44 | -1.337 | 0.377 |
| Intermediate – Late | 0.269 | 2.21 | 0.122 | 0.992 |
| **Larval Mass:** |  |  |  |  |
| Early – Intermediate | -0.00316 | 0.00804 | -0.393 | 0.919 |
| Early – Late | 0.00868 | 0.00799 | 1.086 | 0.524 |
| Intermediate – Late | 0.01184 | 0.00747 | 1.584 | 0.256 |

***Figure S1.*** *Larval brood size in response to the absence or presence of C. vomitoria (A) and to the timing and density of C. vomitoria (according to our 3 × 2 design: B). A. Comparison between fly-absent and fly-present treatments, showing point estimates (mean ± SE) for larval brood size; black points represent treatment means, while grey points indicate individuals. B. Larval brood size across timing (left – right: early, intermediate, late) and fly density (blue: low, 1 fly; orange: high, 10 flies), showing point estimates (mean ± SE); black points represent treatment means, coloured points indicate individuals, while grey indicates the overall mean for that timing treatment. The red dashed line indicates the control fly-absent value, extended from panel A, for reference.*
